# A novel 9 kDa phosphoprotein is a component of the primary cilium and interacts with polycystin-1

**DOI:** 10.1186/2046-2530-1-S1-P74

**Published:** 2012-11-16

**Authors:** DPS Osborn, C Boucher, P Wilson, V Gattone, PL Beales, I Drummond, R Sandford

**Affiliations:** 1 UCL Institute of Child Health, UK; 2 Department of Medical Genetics, University of Cambridge, UK; 3 The Royal Free Hospital, London, UK; 4 Indiana University School of Medicine, USA; 5 Massachusetts General Hospital, USA

## 

Polycystin-1 (PKD1) forms a mechanosensitive cation channel complex with polycystin-2 (PKD2) in the primary cilium. Functional defects in this complex caused by mutation of PKD1 and PKD2 result in autosomal dominant polycystic kidney disease (ADPKD). The mechanisms by which this complex regulates normal cell physiology remain elusive. In particular the proteins that interact directly with polycystin-1 remain poorly characterised. A Y-2H screen using the C-terminus of polycystin-1 as bait identified a novel binding partner that we have called PIP9 (polycystin interacting protein 9). PIP9 is evolutionary conserved, ubiquitously expressed, and predicted to be a 9KDa phosphoprotein containing a single coiled-coil domain that interacts with the coiled-coil domain of PC-1. Specific anti-PIP9 antisera confirmed the widespread and developmentally regulated expression of PIP9 and identified phosphorylated isoforms. PIP9 co-localised to the renal primary cilium with PC1 and immuno-EM studies revealed localisation to IFT particles. Knockdown of *zPip9*, using morpholino oligonucleotides, resulted in developmental defects in gastrulation movements. In addition, evidence of cell detachment was observed, common to the phenotype described for double *bbs4;bbs10* morphant zebrafish which suggests pip9 may act in the same genetic pathway. *zpip9* morphants also displayed variable head and eye degeneration, including coloboma and hydrocephalus, pronephric cysts and shortening of the body axis. Loss of Pip9 function in mice is lethal before embryonic day E11.5. This preliminary analysis suggests that PIP9 plays a central role in mediating polycystin-1 dependant signalling.

